# Phosphorylated Alpha-Synuclein in Red Blood Cells as a Potential Diagnostic Biomarker for Multiple System Atrophy: A Pilot Study

**DOI:** 10.1155/2020/8740419

**Published:** 2020-01-31

**Authors:** Xu-Ying Li, Weiwei Yang, Xin Li, Xu-Ran Li, Wei Li, Qihan Song, Linjuan Sun, Feng Lin, Zhigang Chen, Chaodong Wang, Shun Yu

**Affiliations:** ^1^Department of Neurobiology, Xuanwu Hospital of Capital Medical University, Beijing 100053, China; ^2^Department of Neurology, Xiyuan Hospital, China Academy of Chinese Medical Sciences, Beijing 100091, China; ^3^Department of Neurology, Union Hospital of Fujian Medical University, Fuzhou 350001, China; ^4^Department of Neurology, Dongfang Hospital of Beijing University of Chinese Medicine, Beijing 100078, China; ^5^Department of Neurology, Xuanwu Hospital of Capital Medical University, Beijing 100053, China; ^6^National Clinical Research Center for Geriatric Diseases, Beijing 100053, China

## Abstract

Diagnosis of multiple system atrophy (MSA) remains a challenge, due to the complexity and overlapping of its symptoms with other Parkinsonian disorders. The critical role of alpha-synuclein (*α*-syn) in the pathogenesis of MSA makes it an ideal biomarker for the diagnosis of MSA. Although *α*-syn alterations in cerebrospinal fluid (CSF) and blood plasma have been extensively assessed for the utility in diagnosing MSA, inconsistent results have been obtained, presumably due to the contamination by hemolysis and other confounding factors. In this study, levels of serine 129-phosphorylated *α*-syn (pS-*α*-syn), a major pathologic form of *α*-syn, in red blood cells (RBCs), were measured using ELISA in a Chinese cohort consisting of 107 MSA patients and 220 healthy controls. A significant increase in the levels of pS-*α*-syn in RBCs (pS-*α*-syn-RBC) was observed in MSA patients than in healthy controls (14.02 ± 4.02 ng/mg versus 11.89 ± 3.57 ng/mg; *p* < 0.0001). Receiver operating characteristic curve (ROC) indicated that pS-*α*-syn-RBC discriminated the patients well from the controls with a sensitivity of 80.37% (95% confidence interval (CI): 71.58%–87.42%), a specificity of 88.64% (95% CI: 83.68%–92.51%), and an area under the curve (AUC) of 0.91 (95% CI: 0.87–0.94). The levels of pS-*α*-syn-RBC were negatively correlated with RBD-HK scores and differed between MSA-P and MSA-C subtypes (13.27 ± 1.91 versus 12.19 ± 3.04; *p*=0.025). The difference between subtypes was seen at Hoehn and Yahr stages 3 and 4, and the age at onset (AAO) between 60 and 69 years (*p*=0.016). The results suggest that pS-*α*-syn-RBC is increased in MSA patients and can be used as a potential diagnostic biomarker for MSA.

## 1. Introduction

Multiple system atrophy (MSA) is a progressive neurodegenerative disease that consists of two major pathological subtypes: olivopontocerebellar atrophy (OPCA) and striatonigral degeneration (SND), which are clinically defined as the Parkinsonian (MSA-P) and cerebellar (MSA-C) variants, respectively [1]. In both subtypes, the widespread distribution of *α*-synuclein- (*α*-syn) containing glial cytoplasmic inclusions (GCIs) in association with neurodegenerative changes is a pathological feature for a definite diagnosis of MSA [2]. Clinical diagnosis of possible and probable MSA is based on the presence of clinical core features, including progressive dysautonomia, cerebellar ataxia, and/or Parkinsonism [2]. However, owing to the concomitant pathological changes and overlapping phenotypes with other Parkinsonian disorders such as Parkinson's disease (PD), dementia with Lewy bodies (DLB), and progressive supranuclear palsy (PSP) [3], correct antemortem diagnosis of MSA remains a challenge. Therefore, there is an urgent need for biomarkers able to help to diagnose MSA in clinical practice.

Various molecules, including *α*-syn, DJ-1, tau, A*β*, neurofilament light chain, dopamine and its metabolites, and neuroinflammatory cytokines, have been investigated for their usefulness as biomarkers for the diagnosis of MSA [4–6], among which *α*-syn in body fluids is the most studied, owing to the critical role of this protein in the pathogenesis of MSA [7]. However, results obtained so far in the detection of the total, oligomeric, and phosphorylated *α*-syn in cerebrospinal fluid (CSF) and blood plasma have been inconsistent and variable, with the low diagnostic performance [4–6, 8–13]. The lack of consistency and stability has been attributed to the interference by plasma proteins such as lipoproteins and heterophilic antibodies, contamination by hemolysis, and differences in detection methods [5, 14].

Since 99% of the *α*-syn in whole blood is present in red blood cells (RBCs) [15], the detection of *α*-syn in RBCs is an alternative, which can avoid the interference encountered in detecting CSF and plasma *α*-syn. Recent studies investigating different forms of RBC-derived *α*-syn, including total, oligomeric, phosphorylated, proteinase K-resistant, and other posttranslationally modified *α*-syn, have shown a possibility of the RBC-derived *α*-syn in differentiating PD from healthy controls and predicting the severity and progression of PD. Although the RBC-derived oligomeric *α*-syn displayed moderate diagnostic power for MSA [16], the correlations between total *α*-syn in RBCs and MSA have been controversial [17–19], possibly due to the relatively small sample sizes of the studies. Since serine-129 phosphorylated *α*-syn (pS-*α*-syn) is a dominant pathological modification of *α*-syn in the MSA brain [9, 20], it is intriguing to investigate whether this form of *α*-syn in RBCs (pS-*α*-syn-RBC) can be used as a diagnostic biomarker for MSA. Since pS-*α*-syn is a dominant pathological modification of *α*-syn in the MSA brain [9, 20], it is intriguing to investigate whether pS-*α*-syn-RBC can be used as a diagnostic biomarker for MSA.

In this study, we measured the levels of pS-*α*-syn-RBC in MSA patients and healthy controls using an enzyme-linked immunosorbent assay (ELISA) and assessed the performance of altered pS-*α*-syn-RBC levels in discriminating MSA patients from healthy controls. We further analyzed the correlations of pS-*α*-syn-RBC levels with various clinical variables.

## 2. Materials and Methods

### 2.1. Participants

A total of 107 patients with MSA, which were clinically diagnosed according to the international clinical guideline [2], were recruited between May 2017 and October 2018, from Xuanwu Hospital of Capital Medical University and Dongfang Hospital of Beijing University of Traditional Chinese Medicine. According to the diagnostic criteria, 34 (31.7%) of the patients were classified as “probable” MSA. Patients with the following conditions were excluded: (i) Parkinsonian syndromes resulting from cerebrovascular, hypoxic, traumatic, infectious, metabolic, or systemic diseases affecting the central nervous system (CNS); (ii) PD or Parkinson's plus syndromes excluding MSA, such as DLB, PSP, and corticobasal degeneration (CBD); (iii) ambiguous diagnosis due to uncertain clinical or imaging features. Participants with incomplete or missing demographic and clinical information were excluded from the subtyping and staging analyses. According to the dominant phenotypes, 75 MSA patients were classified as MSA-P or MSA-C subtypes. Control subjects (220 cases) were recruited from Xinjiekou Community Medical Center, Beijing (Table 1). Case and control subjects were matched for age and gender.

The study was approved by the Institutional Review Board and Ethics Committees of the participating hospitals and medical center. Written informed consent was obtained from each participant or their legal guardians before they were included in the study.

### 2.2. Clinical Assessment

For each patient, clinical features including Hoehn and Yahr (H&Y) stage, Unified Multiple System Atrophy Rating Scale (UMSARS), Montreal Cognitive Assessment (MoCA), rapid eye movement sleep behavior disorder questionnaire-Hong Kong (RBDQ-HK), Hamilton Depression Scale (HAMD), and Hamilton Anxiety Scale (HAMA) scores were assessed according to the scaling guidelines. For control subjects, scores reflecting nonmotor and prodromal symptoms of MSA and Parkinsonism were also assessed.

### 2.3. Collection of RBC Samples

10 ml of whole blood from each subject was drawn into an ethylenediaminetetraacetic acid (EDTA) anticoagulant tube (1.8 mg EDTA/mL blood). The blood was allowed to stand at 4–8°C for 30 min before being centrifuged at 4°C, 1,500× g for 15 min. The upper and middle layers, containing plasma and white blood cells, were collected, aliquoted, and stored at −80°C for other uses. The lower layer, containing RBCs, was washed three times with Hank's balanced salt solution (HBSS) (without Ca^2+^ and Mg^2+^, 137.93 mM NaCl, 5.33 mM KCl, 0.34 mM Na_2_HPO_4_, 0.44 mM KH_2_PO_4_, 4.17 mM NaHCO_3_, and 5.56 mM D-Glucose (Dextrose), pH 7.2–7.4), aliquoted, and preserved in a freezer (−80°C). Total RBC protein concentrations were determined using a bicinchoninic acid (BCA) protein assay kit (Pierce Biotechnology, Rockford, IL, USA).

### 2.4. Preparation of pS-*α*-Syn

Recombinant human *α*-syn was expressed by *Escherichia coli* BL21 cells transformed with pET-15b-NACP plasmids and purified sequentially with ion-exchange chromatography, hydrophobic chromatography, and reverse-phase chromatography [21]. pS-*α*-syn standard proteins were prepared from recombinant human *α*-syn according to the method described before [22]. In brief, purified human *α*-syn proteins were phosphorylated by incubating with casein kinase II (New England Biolabs, Ipswich, MA, USA). The resultant pS-*α*-syn proteins were purified with anion exchange chromatography and verified by Western blotting using an anti-pS-*α*-syn antibody and mass spectrometry. The purified pS-*α*-syn proteins were concentrated by ammonium sulfate precipitation.

### 2.5. Western Blot and Immunodepletion

Proteins were separated by 12.5% sodium dodecyl sulfate-polyacrylamide gel electrophoresis (SDS-PAGE) and transferred onto polyvinylidene fluoride (PVDF) membranes (Millipore Corp., Bedford, MA, USA). Membranes were blocked for 1 hour with 5% nonfat milk in Tris-buffered saline containing 0.05% Tween-20 (TBST) and probed with different primary antibodies diluted in 5% nonfat milk in TBST, including rabbit polyclonal anti-pS-*α*-syn (Ser 129) (sc-135638, Santa Cruz Biotechnology, Santa Cruz, CA, USA) (1 : 5,000) and 3D5 mouse monoclonal anti-human *α*-syn antibody (RRID : AB_2315787) (1 : 10,000) [23]. After washing with TBST, the blots were incubated with horseradish peroxidase-conjugated goat anti-mouse or goat anti-rabbit IgG (1 : 5000; Vector Laboratories, Burlingame, CA, USA). Immunoreactive bands were visualized by enhanced chemiluminescence and measured for densitometry with a Versadoc XL imaging apparatus (Bio-Rad). All experiments were conducted in triplicate. To rule out the possibility for the cross-reaction of the anti-pS-*α*-syn antibody with other proteins, the RBC lysates were first incubated at 4°C overnight with different concentrations of the 3D5 antibody conjugated to Protein A Sepharose 4B Fast Flow (P9424, Sigma-Aldrich, St. Louis, MO, USA) to preabsorb the endogenous *α*-syn, including both pS-*α*-syn and non-pS-*α*-syn. Then, the protein A-antibody-*α*-syn complexes were precipitated, and the supernatants were subjected to Western blotting using the anti-pS-*α*-syn antibody.

### 2.6. Enzyme-Linked Immunosorbent Assays (ELISA) for pS-*α*-Syn

The ELISA method for measuring pS-*α*-syn-RBC was established previously [24]. Briefly, a 96-well ELISA plate was coated with nonbiotinylated anti-pS-*α*-syn polyclonal antibody (sc-135638, Santa Cruz Biotechnology, Santa Cruz, CA, USA) in coating buffer (200 mmol/L NaHCO_3_ buffer [pH 9.6]) (0.1 *μ*g/mL). After washing with PBS containing 0.05% Tween-20 (PBST) and blocking with 10% BSA in PBST, 100 *μ*l of RBC samples or pS-*α*-syn protein standards were added to each well and incubated at 37°C for 2 h. After washing with PBST, 100 *μ*l/well of biotinylated 3D5 antibody (1 *μ*g/mL) was added and incubated at 37°C for 2 h. The wells were washed with PBST and incubated at 37°C for 1 h with 100 *μ*l of ExtrAvidin Alkaline Phosphatase (E-2636; Sigma-Aldrich, St. Louis, MO, USA) diluted 1 : 5,000 in blocking buffer. After washing with PBST, 100 *μ*l/well of enzyme-substrate p-nitrophenyl phosphate (pNPP, N1891; Sigma-Aldrich) was added and incubated at 37°C for 30 min. After that, the plate was read at 405 nm using a microplate reader (Multiskan MK3, Thermo Scientific, UT, USA). Concentrations of pS-*α*-syn-RBC were calculated according to the standard curve and expressed as ng of pS-*α*-syn-RBC per mg of total RBC proteins. All samples were tested in triplicate within the same assay and on the same day.

The calibration curve was constructed using a series of WT-*α*-syn and pS-*α*-syn samples with standard concentration. The assay used rabbit polyclonal anti-pS-*α*-syn as a capture antibody and biotinylated 3D5 mouse monoclonal anti-WT-*α*-syn as a detection antibody. Correlation between the standardized protein concentrations and the absorbance at 405 nm measured was analyzed. The lower limit of detection (LLOD) and low limit of quantitation (LLOQ) were determined by measuring the absorbance of samples of 0.01 M PBS or standard samples in triplicate. The data were collected and the mean and standard deviation are calculated from the results and then multiplied by three to provide the LLOD. The accuracy of the assay and reproducibility in the sample matrix were assessed by spike-and-recovery and linearity-of-dilution assessments. Quality control (QC) samples were used in each tested plate. Intra- and intertest variations of tested and QC samples were expressed by the coefficient of variation (CVs) and calculated as follows: standard deviation/average concentration × 100%.

### 2.7. Statistical Analysis

Prior to statistical analyses, we examined the normal distribution for all the continuous variables using the Kolmogorov-Smirnov test. Demographic data, pS-*α*-syn levels, and clinical variables were compared between MSA cases and controls or MSA subtypes by univariate analyses using *T*-test (normal distributions) or Wilcoxon/Mann–Whitney *U* test (non-Gaussian distribution). The Pearson Chi-square followed by Fisher's exact test was performed to compare the distribution of categorical variables across groups. Multivariate linear regression models were used to assess the correlation of the pS-*α*-syn-RBC levels in MSA patients with clinical variables (disease duration, H&Y stages, symptoms, and clinical scores). Multivariate regression models were constructed for MSA diagnosis and subtyping analyses, which use pS-*α*-syn-RBC levels and demographic and/or clinical variables (including disease duration, H&Y stages, and clinical scores) showing statistical significance in the univariate analyses as independent variables. Receiver operating characteristic (ROC) curves were constructed and the area under the curve (AUC) was calculated to evaluate the performance of the biomarker. All statistical analyses were performed using IBM SPSS Statistics® v22.0.0.0 (SPSS Inc., Chicago, IL, 2013), Medcalc (Microsoft), and GraphPad Prism® v6.0 (GraphPad Software Inc., La Jolla, CA, 2009), and *p* values less than 0.05 were regarded as statistically significant.

## 3. Results

### 3.1. Demographic and Clinical Data of the Study Subjects

**Figure 1:**
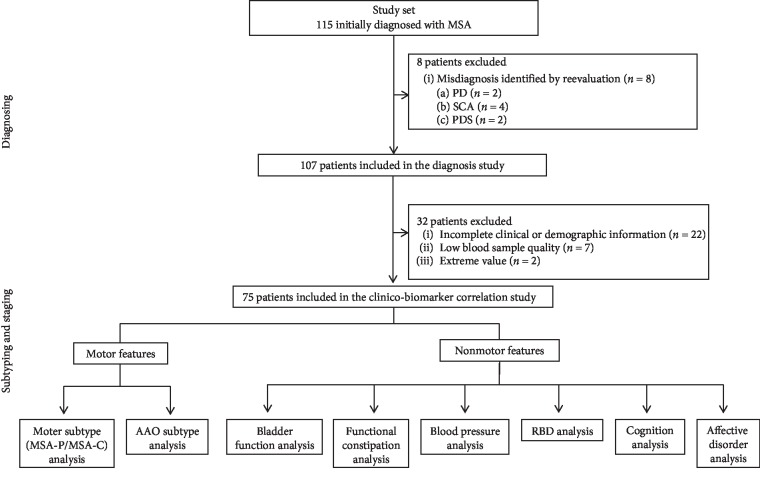
Flow chart of the cohort study.

Between May 2017 and October 2018, 115 potentially eligible MSA patients were recruited, with written consent. After clinical and neuroimaging reevaluation and assurance of blood sample quality, the final participants used in our diagnostic analysis include 107 patients and 220 healthy controls. Among the MSA patients, 75 with complete information underwent subtyping and staging analyses (Figure 1).

Demographic and clinical data for MSA patients and healthy controls are shown in Table 1. The AAO and disease duration of all MSA cases were 58.15 ± 9.85 and 2.93 ± 2.02 years, respectively. Considering subtypes, the AAO and disease duration were 57.24 ± 9.48 and 2.77 ± 2.44 years, respectively, in MSA-P patients, and 58.86 ± 10.18 and 3.06 ± 1.63 years in MSA-C patients. Comparing the clinical scores for motor and nonmotor symptoms (UMSARS I, II, IV, MoCA, RBDQ-HK, HAMD, and HAMA) between the two MSA subtypes by univariate analyses (Mann–Whitney *U* test), the UMSARS Part I scores were significantly higher in MSA-C (18.21 ± 7.82) than in MSA-P (13.67 ± 7.01) patients (*p*=0.022).

### 3.2. Characterization of the ELISA for Detecting pS-*α*-Syn-RBC

**Figure 2:**
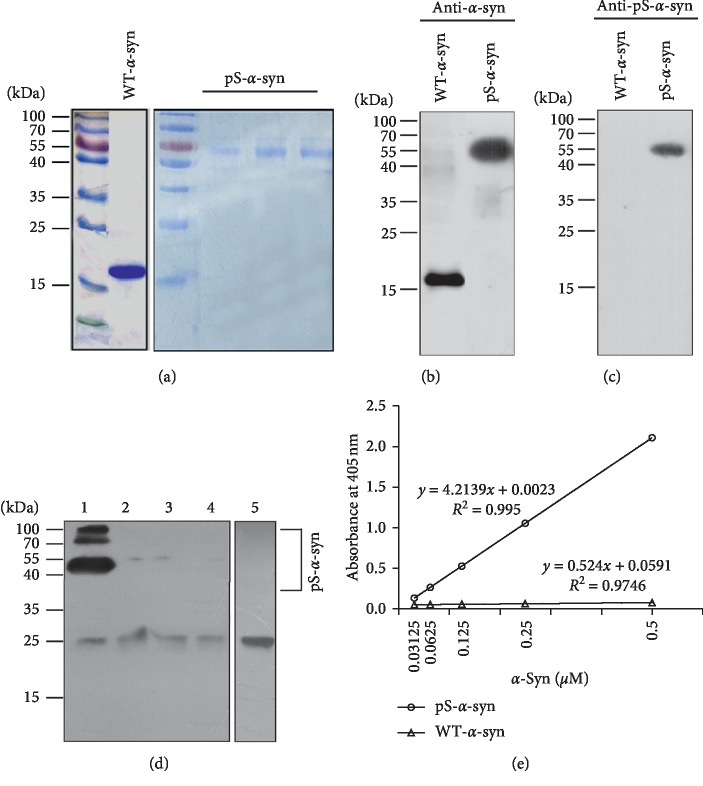
Establishment of the ELISA method for pS-*α*-syn detection. (a) Recombinant human wild type *α*-syn (WT-*α*-syn) and Serine129-phosphorylated *α*-syn (pS-*α*-syn) were purified and validated by Coomassie blue staining and Western blot. (b) and (c) Purified recombinant WT-*α*-syn and pS-*α*-syn were detected by antibodies against WT-*α*-syn (b) and pS-*α*-syn (c), respectively. (d) Immunodepletion test for the specificity of pS-*α*-syn in red blood cell lysates (RBCs). Lane 1: RBC lysates without pre-immunodepleted by the 3D5 antibody; lanes 2–4: RBC lysates pre-immunodepleted by 0.1, 0.33 and 1 *μ*g/ml of the 3D5 antibody. The lysates were analyzed by Western blot using the rabbit anti-pS-*α*-syn as the primary antibody. (e) Calibration curve for the enzyme-linked immunosorbent assay (ELISA) using the anti-pS-*α*-syn as the capture antibody and the biotinylated 3D5 anti-WT-*α*-syn as the detection antibody. Correlations between the standardized protein (WT-*α*-syn and pS-*α*-syn) concentrations and their corresponding absorbance values at 405 nm were plotted and the linear equations and correlation coefficients (R2) were calculated.

The specificity, accuracy, and reproducibility of the ELISA were evaluated using multiple assessments. Coomassie brilliant blue (CBB) staining confirmed a high purity of both the recombinant wild type *α*-syn (WT-*α*-syn) and pS-*α*-syn proteins (Figures 2(a) and 2(b)). Western blot revealed that the 3D5 anti-*α*-syn antibody specifically recognized WT-*α*-syn (17 kDa) and not pS-*α*-syn. In contrast, the anti-pS-*α*-syn antibody specifically bound purified pS-*α*-syn (55 kDa) in a concentration-dependent manner and not WT-*α*-syn (Figure 2(c)). Western blot analysis of the RBC lysates using the anti-pS-*α*-syn antibody revealed several bands, including three major bands ranging from above 55 to 130 kDa and a very weak band at around 28 kDa (Figure 2(d), Lane 1). The three major bands were absorbed by preincubation of the lysates with an overdose of the 3D5 anti-*α*-syn (Figure 2(d), Lane 2–4), where the 28 kDa weak band remained unaffected, indicating that the pS-*α*-syn antibody mainly detects the pS-*α*-syn, which was in an aggregated form. In the ELISA, the absorbance values measured at 405 nm were positively correlated with the standardized pS-*α*-syn concentrations, with an *R*^2^ value of 0.995 (Figure 2(e)). The LLOD and LLOQ of the assay were determined to be 0.18 *μ*g/mL and 0.60 *μ*g/mL, respectively. The spike-and-recovery assessment showed that the recovery rates in the pS-*α*-syn in RBC lysates at the low spike, median spike, and high spike of the standard pS-*α*-syn sample were 87.7%, 90.7%, and 95.9%, respectively (Tables 2 and 3). Linearity-of-dilution test showed that the recovery rates of the 1 : 2, 1 : 4, and 1 : 8 diluent were 119%, 127%, and 138%, respectively (Table 4). The CVs for interassay variation of the triplicate wells for each sample ranged from 0.04% to 0.42% in cases and from 0.07 to 16.19% in control samples. Two quality control (QC) samples were added for the assay, and the CV of the QC samples ranged from 1.54% to 2.60%.

### 3.3. Performance of pS-*α*-Syn-RBC in Diagnosing MSA

**Figure 3:**
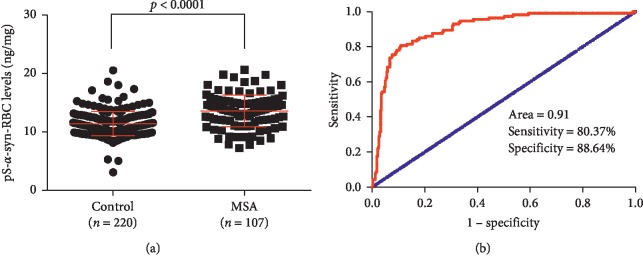
Performance of pS-*α*-syn-RBC in diagnosing MSA. (a) Scatter diagram showing the levels of pS-*α*-syn-RBC in MSA patients and healthy controls. (b) ROC curves showing the diagnostic values of pS-*α*-syn-RBC for MSA. pS-*α*-syn-RBC, serine129-phosphorylated *α*-syn in red blood cells; MSA, multiple system atrophy; ROC, receiver operating characteristic curve; AUC, area under the curve.

The above ELISA was used to measure pS-*α*-syn-RBC. The levels of pS-*α*-syn-RBC were significantly higher in MSA patients (14.02 ± 4.02 ng/mg) than in healthy controls (11.89 ± 3.57 ng/mg) (*p* < 0.001) (Figure 3(a); Table 5). In the multivariate logistic regression model including pS-*α*-syn-RBC level, age, and gender, the AUC of the ROC curve was 0.91 (95% confidence interval (CI): 0.87–0.94), with a sensitivity of 80.37% and a specificity of 88.64% for diagnosing MSA (Figure 3(b)). The cut-off concentration of pS-*α*-syn-RBC for discriminating MSA patients from controls was 10.9 ng/mg. Using this concentration, the likelihood for positively (LR+) predicting MSA was 7.07, while the negatively predicting (LR-) MSA was 0.22 (Table 5). Considering the difference between the clinically and pathologically diagnosed MSA, we also analyzed the pS-*α*-syn-RBC levels in the “probable MSA” cases. As a result, the levels of pS-*α*-syn-RBC of these cases were 12.08 ± 0.36 ng/mg, which was significantly higher than those of controls (*p*=0.021). The sensitivity and specificity of pS-*α*-syn-RBC in discriminating probable MSA cases and controls were 58.67% (95%CI: 46.70%–69.92%) and 74.73% (95%CI: 64.53%–83.25%), respectively, and the AUC was 0.733 for the model discriminating probable MSA patients from controls.

### 3.4. Association of pS-*α*-Syn-RBC with MSA Subtypes

**Figure 4:**
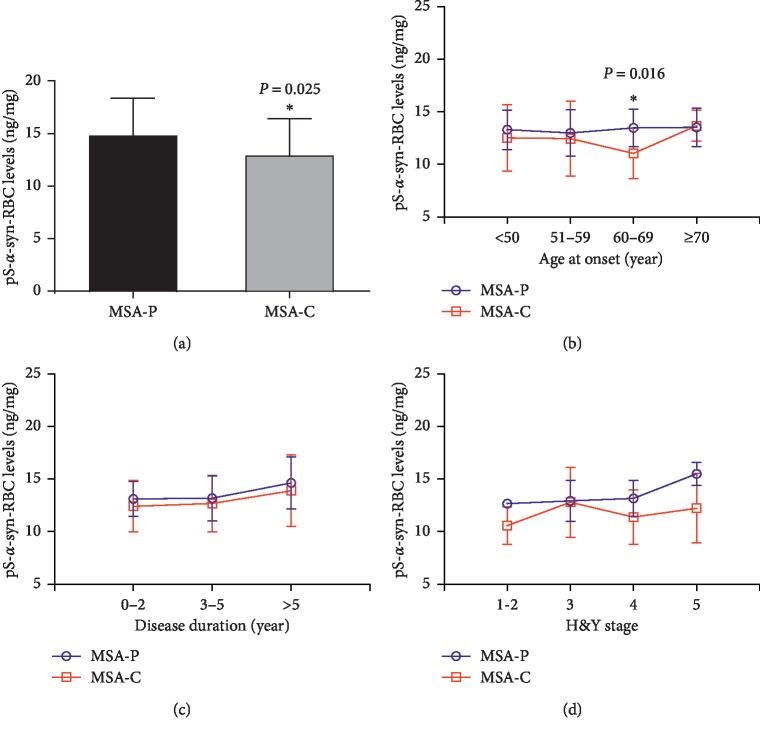
Comparison of pS-*α*-syn-RBC levels between patients with different clinical features. (a) Compared to MSA-C patients, the levels of pS-*α*-syn-RBC in MSA-P patients were significantly higher (adjusted *p*=0.025). (b) The levels of pS-*α*-syn-RBC between MSA-P and MSA-C were only significantly different in patients with the AAO between 60 and 69 years (*p*=0.016). (c) and (d) pS-*α*-syn-RBC levels showed no significant correlation with disease duration (0–2, 3–5, >5 years) or H&Y stage (1-2, 3, 4, 5). MSA, multiple system atrophy; MSA-P, MSA with predominant parkinsonism; MSA-C, MSA with predominant cerebellar ataxia; AAO, age at onset.

**Figure 5:**
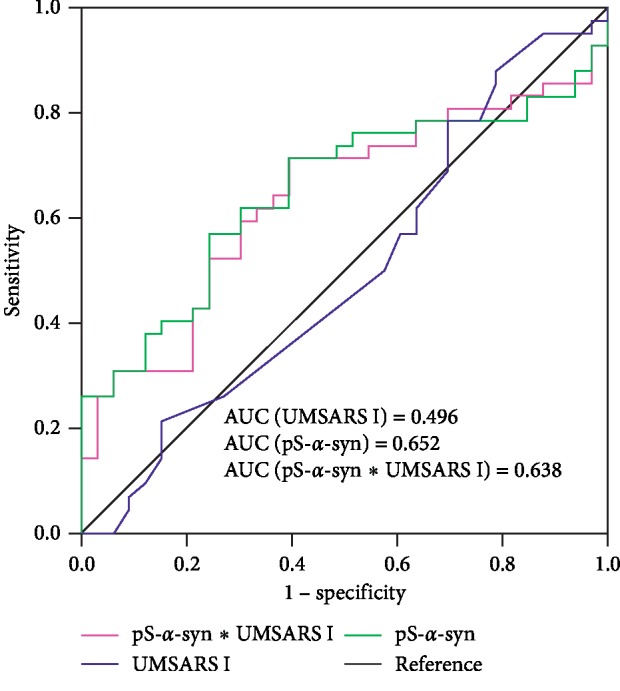
ROC curves for predicting MSA with different subtypes (MSA-P/MSA-C) using pS-*α*-syn-RBC. ROC curves for predicting MSA-P and MSA-C types. Predictive performance was compared between models including UMSARS I score only, the pS-*α*-syn-RBC level only, and the combination of them. UMSARS I: Unified Multiple System Atrophy Rating Scale Part I. The AUC for the model and *p*values for the comparisons between the AUCs included in each analysis are indicated.

Among all the 107 MSA patients, 33 MSA-P patients and 42 MSA-C patients (total 75) had complete clinical information and eligible for subtyping and staging study. The pS-*α*-syn-RBC levels were significantly higher in MSA-P than in MSA-C patients (13.27 ± 1.91 versus 12.19 ± 3.04; *p* = 0.025) (Figure 4(a), Table 6). The difference between subtypes was significant in patients with the AAO ranging from 60 to 69 years (*p* = 0.016; Figure 4(b), Table 6). The difference between subtypes was also seen in patients with H&Y stages 4 and 5, although it was not statistically significant (*p* > 0.05) (Figure 4(c), Table 6). In addition, pS-*α*-syn-RBC levels were observed to play more important role in distinguishing MSA subtypes than UMSARS I score or the combination (AUC_[pS-*α*-syn-RBC]_ = 0.652 (95% CI: 0.526–0.777); AUC_[UMSARS I]_ = 0.496 (95% CI: 0.362–0.631); AUC_[pS-*α*-syn-RBC∗UMSARS I]_ = 0.638 (95% CI: 0.511–0.764), Figure 5).

### 3.5. Correlations between pS-*α*-Syn-RBC Levels and Clinical Features of MSA Patients

**Figure 6:**
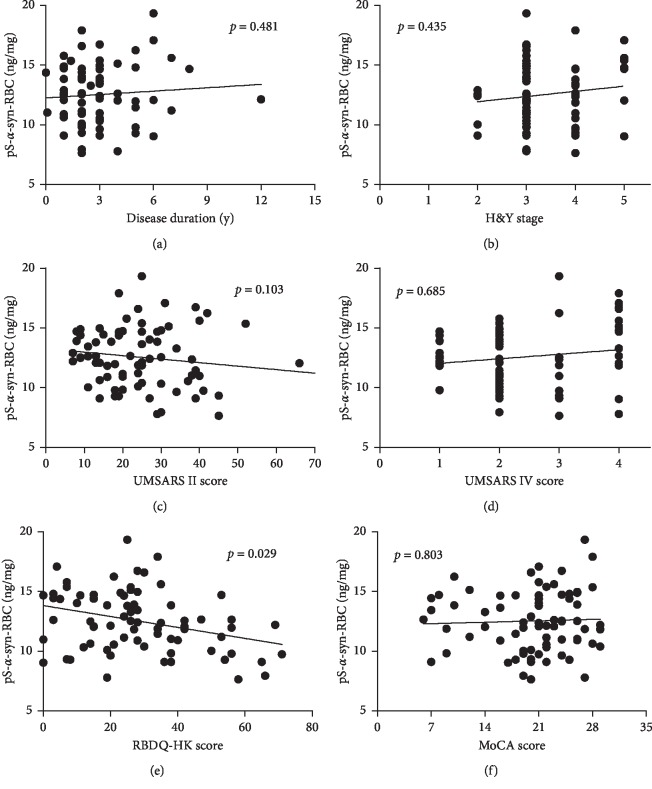
Correlations between pS-*α*-syn-RBC and scores for motor and nonmotor symptoms of MSA. Correlations between pS-*α*-syn-RBC levels and scores for motor and nonmotor symptoms were investigated using a multivariate linear regression model adjusting age, gender, and/or disease duration. Disease duration (a); H&Y stage (b); UMSARS II, Unified Multiple System Atrophy Rating Scale part II (c) and IV (d); RBDQ-HK, Rapid eye movement (REM) sleep behavior disorder questionnaire-Hong Kong (e); MoCA, Montreal Cognitive Assessment (f).

To investigate the association between pS-*α*-syn-RBC and clinical features of MSA patients, we first compared the pS-*α*-syn-RBC levels in patients with and without each nonmotor symptom by univariate analysis (Mann–Whitney *U* test, Table 7). As a result, the pS-*α*-syn-RBC levels were not significantly different between patients with and without these symptoms. In multivariate linear regression analyses using pS-*α*-syn-RBC level as a dependent variable, individual clinical parameter (disease duration, H&Y stage, etc.) as independent variables, and age, gender, and disease duration as covariates, a negative correlation was revealed between pS-*α*-syn-RBC levels with RBDQ-HK scores in MSA patients (*R*^2^ = 0.031, *p*=0.029). However, no significant correlations were observed between pS-*α*-syn-RBC levels and the disease duration, H&Y stage, UMSARS (II and IV), or MoCA scores (all *p* > 0.05, Figure 6 and Table 8).

## 4. Discussion

The ELISA assay used for measuring pS-*α*-syn-RBC was established previously [25] and has been applied to detect pS-*α*-syn in aging monkey brains [24–26] and pS-*α*-syn formed in PD plasma [27]. The detection antibody was 3D5 mouse monoclonal anti-*α*-syn, which recognizes a sequence of 115–121 amino acids specific to human *α*-syn [23] and has been demonstrated previously for its specificity in detecting *α*-syn in human RBCs [16]. The capture antibody was a rabbit polyclonal antibody. The specificity of this anti-pS-*α*-syn in detecting pS-*α*-syn in human RBCs was confirmed by immunodepletion experiments, in which the immunoreactive signals revealed by this antibody in RBC lysates were largely disappeared after the lysates were preincubated with the 3D5 anti-*α*-syn antibody, which could absorb both the WT-*α*-syn and pS-*α*-syn. In order to further characterize our ELISA assay, we performed the spike-and-recovery and linearity-of-dilution assessments as well as LLOD and LLOQ analyses. The results, together with those from the immunodepletion experiments, suggested enough sensitivity, specificity, accuracy, and reproducibility of this assay in detecting pS-*α*-syn in human RBCs.

A recent study reported the presence and increase of pS-*α*-syn in the RBCs of PD patients [18]. In the present study, by measuring the levels of pS-*α*-syn-RBC in a Chinese cohort consisting of 107 MSA patients and 220 healthy controls, we provide evidence that the pS-*α*-syn-RBC in MSA patients was also significantly increased in comparison to that in healthy controls. Using pS-*α*-syn-RBC as a biomarker, the MSA patients were separated well from the healthy controls, with a sensitivity of 80.37%, a specificity of 88.64%, and an AUC of 0.91. The diagnostic values for MSA obtained using pS-*α*-syn-RBC were better than those obtained by detecting any species of plasma- and CSF-derived *α*-syn [4–6, 8–13] and by measuring the total and oligomeric *α*-syn in RBCs [16, 18, 19]. The high performance of pS-*α*-syn-RBC in MSA diagnosis can be explained not only by the lack of the interfering factors encountered in detecting plasma *α*-syn [5, 14], but also by the close relevance of pS-*α*-syn to the neuropathology of MSA, since this type of *α*-syn is a dominant pathological modification of *α*-syn in familial and sporadic Lewy body disease including PD, DLB, and MSA [20]. Although the direct link between RBC- and brain-derived *α*-syn remains to be elucidated, recent studies have shown a high consistency between *α*-syn changes in the two sites. For example, the levels of the total, oligomeric, and phosphorylated *α*-syn were shown to be elevated in the RBCs of PD patients [16, 18], and these forms of *α*-syn were reported to be also increased in the brain of PD patients [20, 28, 29]. Two potential mechanisms may affect the level of pS-*α*-syn in the RBCs of PD patients. One is the altered enzymes regulating *α*-syn phosphorylation in the RBCs. This could be confirmed by measuring some key enzymes in RBCs, such as polo-like kinase 2 (PLK2), an enzyme that promotes *α*-syn phosphorylation, and protein phosphatase 2A, an enzyme that facilitates *α*-syn dephosphorylation [24]. The two enzymes have been reported to be changed in the plasma of PD patients [25]. Whether these enzymes are also changed in the RBCs of PD patients remains to be investigated. In addition, the levels of pS-*α*-syn in RBCs may be influenced by brain pS-*α*-syn concentration. There is evidence that *α*-syn can be secreted from neuronal cells by exocytosis [30, 31] and transported across the blood-brain barrier (BBB) in the brain-to-blood direction [32, 33]. This means that the brain pS-*α*-syn can be released into the blood plasma. Whether plasma *α*-syn can be further translocated into RBCs is a subject that needs further exploration.

In addition to separating MSA patients from healthy controls, the levels of pS-*α*-syn-RBC were different between subtypes of MSA. Compared with the MSA-C subtype, patients with MSA-P subtype had higher levels of pS-*α*-syn-RBC, which was especially prominent in those with the AAO between 60–69 years. Moreover, higher levels of pS-*α*-syn-RBC were apparently seen in MSA-P patients with H&Y stages 4 and 5 in comparison to MSA-C patients with the same H&Y stages, although the difference was not statistically significant. The mechanisms for the above difference are not clear. Potential reasons might include the following: (i) the limited number of cases in this study; (ii) the potential bias in H&Y staging, where the patients with MSA-C, who present with cerebellum ataxia and gait imbalance in early stage, usually reach higher H&Y stage while their *α*-syn pathology may be still in a relatively early stage; and (iii) the mean disease duration which was shorter in MSA-P than in MSA-C subtypes [33], indicating that MSA-P subtype may have a more severe overload and faster propagation of *α*-syn pathology than MSA-C subtype. In addition to the above observations, this study showed that the levels of pS-*α*-syn-RBC were negatively correlated with RBDQ-HK scores, an important nonmotor symptom for MSA. The reason for this is not clear. Although RBD is a risk factor for synucleinopathies including MSA [34], the mechanism may be more complicated, and other factors in addition to *α*-syn accumulation may participate in the formation of RBD.

One major limitation of the study was the lack of comparison of pS-*α*-syn-RBC levels between patients with MSA and other neurodegenerative disorders such as PD, DLB, and PSP. Since the recruited patients were clinically diagnosed MSA patients using the current consensus criteria, the diagnostic accuracy remains a challenge. According to a retrospective autopsy study in a cohort of patients with a clinical diagnosis of MSA, only 62% of patients with a clinical diagnosis of MSA had their diagnosis confirmed at autopsy [35]. In addition, our results showed that the performance (sensitivity: 58.67%; specificity: 74.73%; AUC: 0.733) of using this marker for discriminating probable MSA from controls was poorer than the overall case-control analyses, suggesting that this marker might be more sensitive and specific for definite MSA than for probable MSA. Thus, further studies are needed to evaluate the possibility and usefulness of pS-*α*-syn-RBC alone, or in combination with other biomarkers in the differential diagnosis between distinct neurodegenerative movement disorders. In addition, other factors (e.g., RBC counts) that may affect the accuracy of the detection methods used in our study need to be further evaluated.

In summary, the present study provides evidence that pS-*α*-syn-RBC can be used as a potential diagnostic biomarker for discriminating MSA patients from healthy controls. In addition, the present study demonstrates a difference between MSA subtypes for the level of pS-*α*-syn-RBC. However, our current results cannot determine whether pS-*α*-syn-RBC can be used for early diagnosis of MSA. Longitudinal studies using large cohorts consisting of patients at different stages (prodromal, early, and advanced) are needed to address this possibility.

## Figures and Tables

**Table 1:** Demographic data, pS-*α*-syn-RBC level, and clinical characteristics of the study subjects.

Variables	Control	Total MSA	*p* values	MSA-P	MSA-C	*p* values
*Demographic data*						
No.	220	107	NA	48	59	NA
Gender (male/female)	127/93	61/46	0.512^c^	27/21	30/29	0.494^c^
Age (years)	66.67 ± 10.99	67.49 ± 8.72	0.918^a^	60.03 ± 9.65	61.90 ± 10.61	0.439^a^
**pS-α-syn-RBC level (ng/mg)**	11.89 ± 3.57	14.02 ± 4.02	*p* < 0.001^b^	13.27 ± 1.91	12.19 ± 3.04	*p*=0.025^b^

*Clinical features*						
No.	91	75	NA	33	42	NA NA
Education (years)	10.69 ± 2.95	9.91 ± 4.73	0.221^a^	8.33 ± 4.52	11.15 ± 4.57	**0.009** ^a^
Age at onset (AAO, years)	NA	58.15 ± 9.85	NA	57.24 ± 9.48	58.86 ± 10.18	0.485^a^
Disease duration (years)	NA	2.93 ± 2.02	NA	2.77 ± 2.44	3.06 ± 1.63	0.076^b^
H&Y (median, 25%, 75%)	NA	3 (3, 4)	NA	3 (3, 4)	3 (3, 4)	0.819^c^
UMSARS I	1.00 ± 1.77	16.21 ± 7.77	**<0.001** ^b^	13.67 ± 7.01	18.21 ± 7.82	**0.022** ^b^
UMSARS II	0.26 ± 0.66	25.13 ± 13.53	**<0.001** ^b^	25.61 ± 15.64	24.76 ± 11.79	0.791^b^
UMSARS IV (median, 25%, 75%)	1 (1, 1)	2 (2, 3)	**<0.001** ^c^	2 (2, 2)	2 (2, 3)	0.288^c^
MoCA score	25.65 ± 2.59	20.27 ± 5.92	**<0.001** ^b^	19.61 ± 6.16	20.79 ± 5.75	0.589^b^
AHRS score	23.57 ± 1.85	22.93 ± 3.06	0.091^b^	23.27 ± 2.27	22.67 ± 3.56	0.796^b^
RBDQ-HK score	4.81 ± 4.30	28.23 ± 17.76	**<0.001** ^b^	31.10 ± 17.47	25.81 ± 17.82	**0.013** ^b^
HAMD score	1.77 ± 2.25	9.55 ± 6.09	**<0.001** ^b^	9.95 ± 6.17	9.95 ± 6.11	0.860^b^
HAMA score	4.02 ± 3.67	7.17 ± 6.05	**<0.001** ^b^	8.09 ± 5.91	6.45 ± 6.13	0.118^b^

All data are expressed as mean ± SD. Bold: *p* < 0.05. ^a^*T*-test; ^b^Mann-Whitney *U* test; ^c^Chi-square test. MSA, multiple system atrophy; MSA-P, MSA with predominant parkinsonism; MSA-C, MSA with predominant cerebellar ataxia; UMSARS, Unified Multiple System Atrophy Rating Scale; H&Y stage, Hoehn and Yahr stage; MoCA, Montreal Cognitive Assessment; AHRS, Hyposmia Rating Scale; RBDQ-HK, rapid eye movement (REM) sleep behavior disorder questionnaire-Hong Kong; HAMD, Hamilton Depression Scale; HAMA, Hamilton Anxiety Scale; NA, not applicable.

**Table 2:** ELISA spike-and-recovery assessments of pS-*α*-syn in RBC lysates.

Sample	No spike (0 *μ*g/ml)	Low spike (1.5 *μ*g/ml)	Medium spike (3.0 *μ*g/ml)	High spike (4.5 *μ*g/ml)
0.01 M PBS	0	1.70	2.80	4.00
RBC1	1.35	1.45	2.38	3.81
RBC2	1.13	1.56	2.87	3.65
RBC3	2.07	1.41	2.57	3.98
RBC4	1.64	1.59	2.39	3.82
RBC5	1.91	1.46	2.48	3.96
RBC6	2.23	1.48	2.46	3.83
RBC7	1.86	1.51	2.72	3.75
RBC8	1.77	1.49	2.59	3.77
RBC9	1.42	1.47	2.36	3.98

Samples were assayed by adding 100 *μ*l of sample and a different spike stock solution to yield the intended 0, 1.5, 3, or 4.5 *μ*g/ml spike concentration. Values reported for spike samples reflect a subtraction of the endogenous (no spike) values. Recoveries for spiked test samples were calculated by comparison to the measured recovery of spiked diluent. All values represent the average of three replications.

**Table 3:** Typical presentation for summarizing spike-and-recovery results.

Sample (*n*)	Spike level	Expected	Observed	Recovery (%)
RBC (9)	Low (1.5 *μ*g/ml)	1.7	1.49	87.7
	Med (3.0 *μ*g/ml)	2.8	2.54	90.7
	High (4.5 *μ*g/ml)	4.0	4.27	95.9

**Table 4:** ELISA linearity-of-dilution results for the tested RBC sample.

Dilution factor (DF)	Observed (*μ*g/ml) × DF	Expected (*μ*g/ml, neat value)	Recovery (%)
Neat	2.6	2.6	100
1 : 2	3.1		119
1 : 4	3.3		127
1 : 8	3.6		138

Dilutions were made in a previously chosen sample diluent. The observed value was assessed relative to the assay standard curve.

**Table 5:** Statistics for pS-*α*-syn-RBC in diagnosis and subtyping analyzing of MSA.

Comparison	pS-*α*-syn-RBC (ng/mg, Mean ± S.D)	AUC (95% CI)	*p*-value	Cut-off^*∗*^ (ng/mg)	Sensitivity (%, 95% CI)	Specificity (%, 95% CI)	PPV (%, 95%CI)	NPV (%, 95% CI)	LR (+)†	LR (−)‡
MSA/Control	14.02 ± 4.02/11.89 ± 3.57	0.91 (0.87–0.94)	**<0.001**	10.90	80.37 (71.58–87.42)	88.64 (83.68–92.51)	81.87 (74.65–87.99)	87.62 (81.43–92.10)	7.07	0.22
MSA-P/MSA-C	13.27 ± 1.91/12.19 ± 3.04	0.65 (0.53–0.78)	**=0.025**	12.08	57.14 (40.96–72.28)	75.76 (57.74–88.91)	63.87 (49.44–76.23)	70.21 (49.22–86.70)	2.36	0.57

^*∗*^The cur-off values were determined by Youden's suggested index. †LR (+) = sensitivity/1-specificity. ‡NPV = LR (−) = 1-sensitivity/specificity. Bold: *p* < 0.05. pS-*α*-syn-RBC, phosphorylated *α*-syn in RBC; AUC, area under curve; PPV, positive predictive value; NPV, negative predictive value; LR (+), positive likelihood ratio; LR (−), negative likelihood ratio.

**Table 6:** pS-*α*-syn-RBC levels of the study subjects.

pS-*α*-syn levels in RBCs	MSA-total (*n* = 75)	MSA-P (*n* = 33)	MSA-C (*n* = 42)	*p* values
All	12.67 ± 2.64	13.27 ± 1.91	12.19 ± 3.04	**0.025** ^a^
*pS-α-syn-RBC levels in patients stratified by age at onset, ng/mg*				
<50	12.75 ± 2.80	13.30 ± 1.88	12.53 ± 3.15	0.658
≥50	12.64 ± 2.63	13.27 ± 1.94	12.08 ± 3.04	**0.050** ^a^
50–59	12.70 ± 3.00	12.99 ± 2.20	12.46 ± 3.58	0.645
60–69	12.15 ± 2.44	13.47 ± 1.80	11.04 ± 2.40	**0.016**
≥70	13.59 ± 1.61 (*p* for trend: 0.564)	13.53 ± 1.85 (*p* for trend: 0.926)	13.68 ± 1.46 (*p* for trend: 0.416)	0.897

*pS-α-syn-RBC levels in patients stratified by disease duration, ng/mg*				
0–2	12.41 ± 2.43	13.11 ± 1.65	11.86 ± 2.81	0.111
3–5	12.68 ± 2.70	13.17 ± 2.14	12.25 ± 3.12	0.406
>5	13.90 ± 3.39 (*p* for trend: 0.474)	14.63 ± 2.47 (*p* for trend: 0.528)	13.46 ± 4.05 (*P* for trend: 0.609)	0.670

*pS-α-syn-RBC levels in patients stratified by H&Y stages, ng/mg*				
1–2	11.41 ± 1.72	12.66 ± 0.35	10.57 ± 1.79	0.217
3	12.85 ± 2.75	12.92 ± 1.96	12.79 ± 3.31	0.649^a^
4	12.04 ± 2.42	13.16 ± 1.70	11.38 ± 2.60	0.125
5	14.09 ± 2.69 (*p* for trend: 0.215)	15.49 ± 1.10 (*p* for trend: 0.092)	12.23 ± 3.28 (*p* for trend: 0.539)	0.116

^a^Mann–Whitney *U* test.

**Table 7:** Comparisons of pS-*α*-syn-RBC levels in MSA patients with and without nonmotor symptoms.

Symptoms	No	Yes	*p* values
Bladder dysfunction	12.00 ± 2.61	13.00 ± 2.62	0.126
Functional constipation	12.19 ± 2.38	13.35 ± 2.88	0.060
Orthostatic hypotension	13.95 ± 2.80	12.40 ± 2.55	0.054
REM behavior disorder	12.88 ± 2.27	12.57 ± 2.80	0.645
Cognition impairment	12.71 ± 2.78	12.62 ± 2.54	0.877
Affective disorders	12.67 ± 2.37	12.66 ± 2.84	0.997

**Table 8:** Beta-coefficients and 95% confidence interval for multivariate linear regression analysis.

Symptoms	B	Normalized *β*	95% CI	
			Lower	Higher
Duration	0.116	0.088	−0.210	0.442
H&Y	0.335	0.093	−0.515	1.185
UMSARS II	−0.038	−0.193	−0.083	0.008
UMSARS IV	0.130	0.049	−0.509	0.770
RBDQ-HK	−0.040	−0.269	−0.076	−0.004
MoCA	0.013	0.030	−0.093	0.120

## Data Availability

The data we used are from quantification analysis from blood testing. All these data used to support the findings of this study are included within the article and can be shared with anyone.
